# Evaluating the Effectiveness of Skills Training in Affective and Interpersonal Regulation (STAIR) Therapy for Complex Post Traumatic Stress Disorder Delivered by Core Psychiatry Trainees

**DOI:** 10.1192/bjo.2024.455

**Published:** 2024-08-01

**Authors:** Dasal Abayaratne

**Affiliations:** Sheffield Health and Social Care NHS Foundation Trust, Sheffield, United Kingdom

## Abstract

**Aims:**

This project aims to evaluate the effectiveness of Skills Training in Affective and Interpersonal Regulation (STAIR) psychotherapy delivered by Core Psychiatry Trainees (CPTs) within the Sheffield Specialist Psychotherapy Service; a regional tertiary psychotherapy service for people with complex trauma and personality difficulties.

STAIR is a manualised evidence-based skills-based psychotherapy for people with Complex Post Traumatic Stress Disorder (cPTSD) awaiting trauma processing that is deliverable by a range of qualified and non-qualified staff. It was introduced to address two key difficulties the service faces: a long waiting list for trauma processing potentially contributes to patient deterioration, and a difficulty in identifying suitable cases for CPT short psychotherapy case requirements given the majority of potential patients awaited longer term psychotherapy.

**Methods:**

A modified STAIR protocol was developed to meet the requirements of CPTs.

A 1-year prospective evaluation was used to compare pre and post patient reported outcome measures. These include the Nine item Patient Health Questionnaire (PHQ9) for depression symptoms, Impacts of Events Scale Revised (IES-R) for trauma symptoms, Recovering Quality of Life – 10 question (ReQoL-10) for quality of life, and the Short form Inventory of Interpersonal Problems (IIP-32) for relational symptoms. Descriptive statistics were used and data analysed using repeated measure t-tests.

**Results:**

17 patients completed STAIR delivered by CPTs. There was statistically significant mean improvement in Quality of Life (p = 0.001), trauma symptoms (p = 0.009) and depression symptoms (p = 0.019). Mean ReQoL-10 and IES-R improvements additionally met criteria for reliable change. There was non-significant (p = 0.0146) improvement in relational symptoms measured by IIP-32.

**Conclusion:**

This evaluation demonstrates promising patient outcomes from STAIR delivered by CPTs for people with Complex PTSD awaiting trauma processing. This may help both negate any potential deteriorations whilst awaiting therapy, as well as prepare patients. Further evaluations could focus on acceptability and outcomes for CPTs.

Whilst the nature of this small evaluation limits further interpretation and generalisability, this pathway offers a promising means of meeting CPT psychotherapy competencies whilst also improving outcomes for patients.

